# Proximal Ulna Adamantinoma

**DOI:** 10.1016/j.jhsg.2025.100899

**Published:** 2025-12-05

**Authors:** Liam H. Wong, Rosanna Wustrack, Nicolas Lee, Leah Demetri

**Affiliations:** ∗ Department of Orthopaedic Surgery, University of California San Francisco, San Francisco, CA; † Department of Orthopaedic Surgery, Mass General Brigham, Boston, MA

**Keywords:** Adamantinoma, One-bone forearm, Osteofibrous dysplasia, Ulnar adamantinoma, Ulna salvage procedure

## Abstract

Adamantinoma is a rare, malignant tumor that is typically seen in the tibia but has been reported in all long bones. We present the case of a woman who presented as a teenager with a pathologic fracture of the proximal ulna that was initially diagnosed as osteofibrous dysplasia and treated with internal fixation. After the lesion was identified in adulthood as adamantinoma, she was converted to a one-bone forearm procedure as a salvage treatment.

Adamantinoma is a rare, slow-growing malignant tumor arising from epithelial cells, typically presenting in individuals aged 20−50 years.^1^ The benign counterpart, osteofibrous dysplasia, is a fibro-osseous lesion with similar radiographic and histologic features and occurs almost exclusively in children.^1^ Distinguishing between these lesions is critical because adamantinoma metastasizes in 20% to 30% of cases and can be fatal, whereas osteofibrous dysplasia never progresses after skeletal maturity.

Adamantinoma occurs almost exclusively in the tibial diaphysis (80% to 90% of cases);^1^ however, cases have been reported in all long bones.^2^^,^^3^ To our knowledge, only 11 ulnar adamantinoma cases have been described.^4^, ^5^, ^6^, ^7^ We present a young woman with ulnar adamantinoma initially diagnosed as osteofibrous dysplasia and describe the utility of the one-bone forearm procedure as salvage treatment using a novel technique of compression screws and a mesh plate contoured to the patient’s specific anatomy.

## Case Report

**Figure 1:**
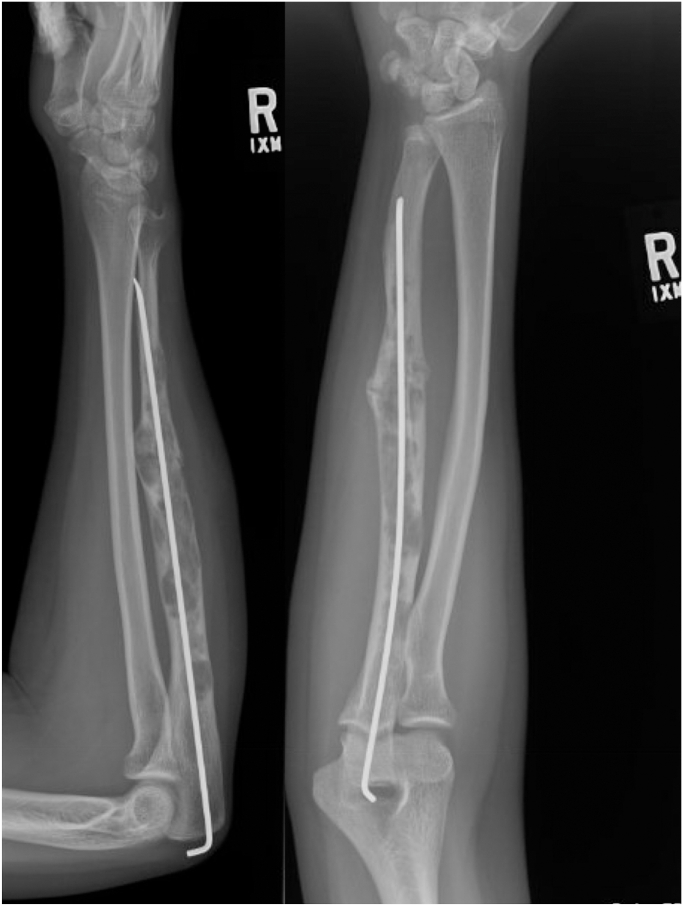
Patient at age 15. One-month postoperative radiograph status post right ulna open biopsy and intramedullary nailing for pathologic fracture.

A 15-year-old right-handed girl presented with a pathologic fracture of the right ulna. She denied antecedent pain prior to fracture and was neurovascularly intact. Radiographs demonstrated a heterogeneous, expansile diaphyseal lesion. Open biopsy demonstrated osteofibrous dysplasia on frozen section and permanent pathological analysis. The fracture was stabilized with an antegrade medullary Steinmann pin (Fig. 1).

Three weeks after surgery, she presented with new abdominal pain. She was diagnosed with a left ovarian malignant germ cell tumor and underwent salpingo-oophorectomy, chemotherapy, and subsequent laparoscopic tumor resection with pathology consistent with teratoma. At 6 months, she had olecranon bursitis because of pin migration with subsequent implant removal. She had continued aching forearm pain that improved with Non-Steroidal Anti-Inflammatory Drugs and was managed conservatively for 11 years.

**Figure 2:**
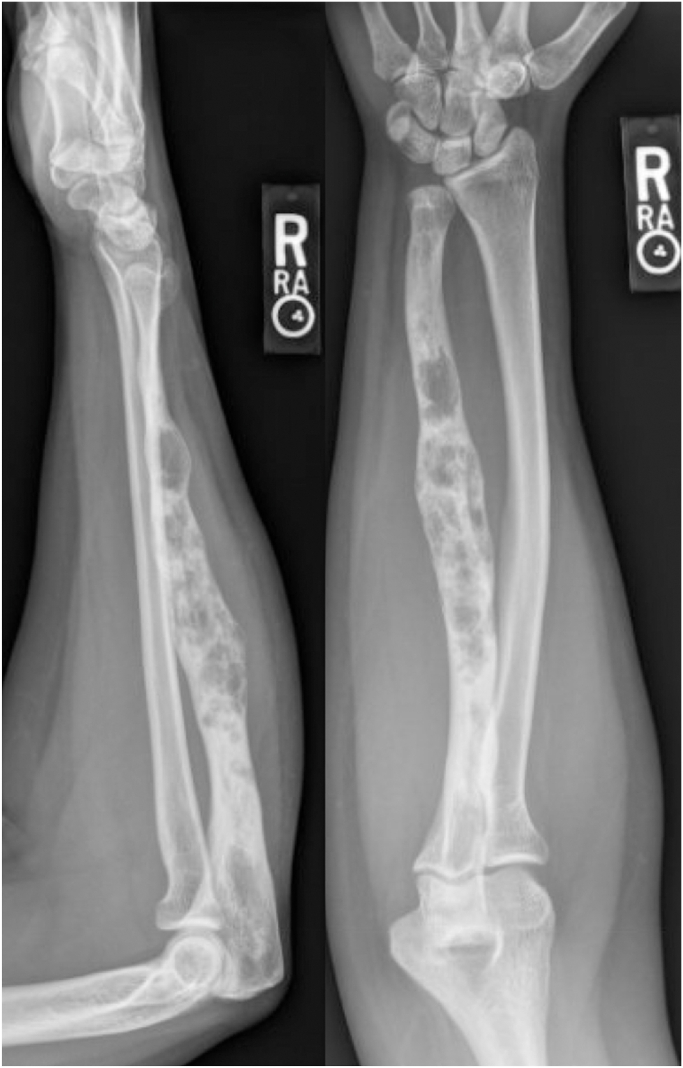
Patient at age 26. Radiographs demonstrated an enlarged ulnar shaft with a mixed lytic and blastic lesion involving greater than 90% of the cortical width.

**Figure 3:**
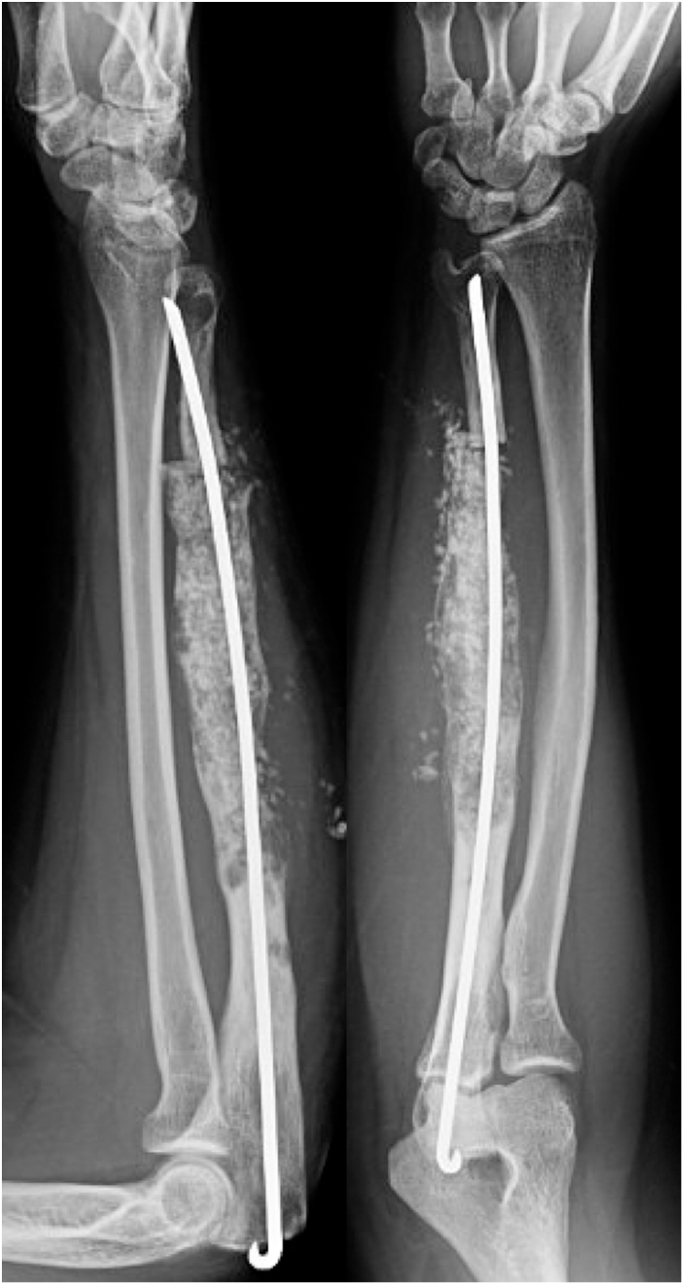
Two months status post right ulna curettage and internal fixation, given intraoperative frozen pathology consistent with osteofibrous dysplasia.

**Figure 4:**
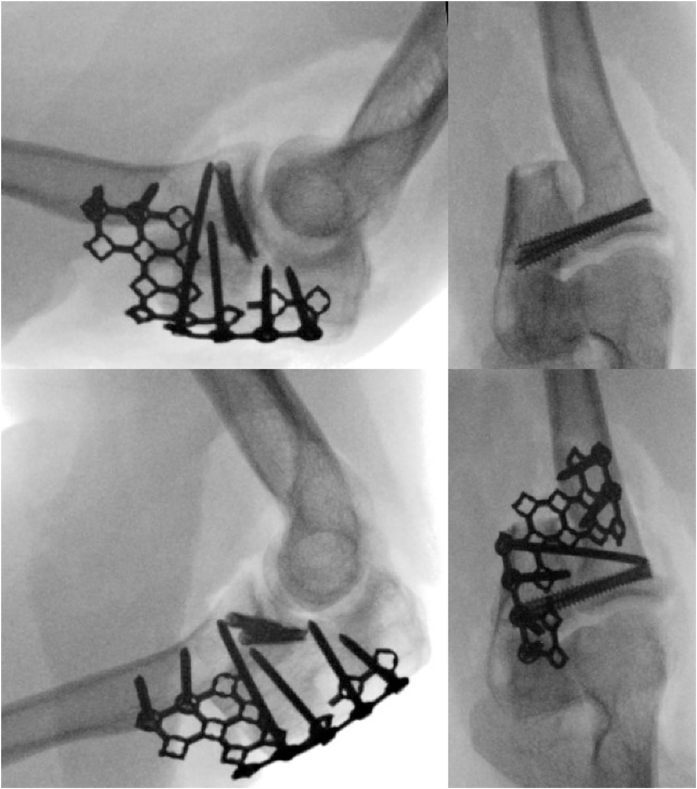
Intraoperative fluoroscopy of the one-bone forearm reconstruction.

At age 26 years, she presented with worsening forearm pain. Radiographs demonstrated an enlarged ulnar shaft with a mixed lytic and blastic lesion involving >90% of the cortical width (Fig. 2). Biopsy was again consistent with osteofibrous dysplasia. The lesion was curetted, irrigated with hydrogen peroxide, and packed with allograft. A distal ulna osteotomy was made and stabilized with a Rush rod (Fig. 3). Permanent pathology demonstrated adamantinoma rather than osteofibrous dysplasia. Staging studies showed no metastases. Given the new malignant adamantinoma diagnosis, a wide resection and reconstruction were performed. A two-stage resection and reconstruction ensured that the remaining proximal ulna was tumor-free. The initial one-bone forearm procedure used headless compression screws at the decorticated proximal radioulnar joint (PRUJ) supplemented with iliac crest cancellous bone autograft and a contourable mesh neutralization plate (Fig. 4). At 2 years, she re-presented with continued elbow pain and radiographic evidence of nonunion with hardware failure requiring revision. All procedures followed were in accordance with the ethical standards of the responsible committee on human experimentation (institutional and national) and with the Helsinki Declaration of 1975, as revised in 2008. Written informed consent was obtained from the patient for publication of this case report and accompanying images.

### Stage 1: Wide resection

The prior approach to the ulna was used. Fluoroscopy was used to confirm the level of the proximal ulna osteotomy, which preserved 5 cm of proximal ulna. An oscillating saw followed by a Gigli saw was used to cut the bone down to the rod, and the rod was cut. The ulna distal to the osteotomy was elevated of the wound with a thin layer of muscle and intraosseous membrane attached to ensure wide margins. The distal radioulnar and ulnocarpal ligaments were transected to remove the ulna. The pin was removed retrograde. A trephine was used to core out the bone around the proximal pin site in the ulna, and the core of bone was sent for permanent pathological analysis. Additional margins were sent for permanent pathological analysis, and bone wax was placed in the canal for hemostasis. A drain was placed, and the wound was closed in a layered fashion. After surgery, margins were negative for residual tumor, and so the decision was made to proceed with a one-bone forearm reconstruction.

### Stage 2: One-bone forearm reconstruction

The prior incision over the posterior border of the ulna was extended proximally to the olecranon. The Kaplan approach between extensor digitorum communis and extensor carpi radialis brevis was used. Taking care to protect the lateral ulnar collateral ligament, a capsulotomy was made, allowing access to the PRUJ. The radial head was dislocated, and the radius and ulna at the PRUJ was decorticated with a burr to cancellous bone. The PRUJ was reduced, and the forearm was rotated to 30° of pronation per patient preference. The PRUJ was held provisionally with two Kirschner wires. Two 4-mm cannulated, variable thread pitch headless compression screws were placed over the guidewires. Iliac crest bone autograft was placed between the radius and ulna just distal to the PRUJ, with additional cancellous graft placed at the level of the PRUJ. A mesh plate was cut, contoured, and secured with 2.7 mm screws (Fig. 4).

### One-bone forearm revision

**Figure 5:**
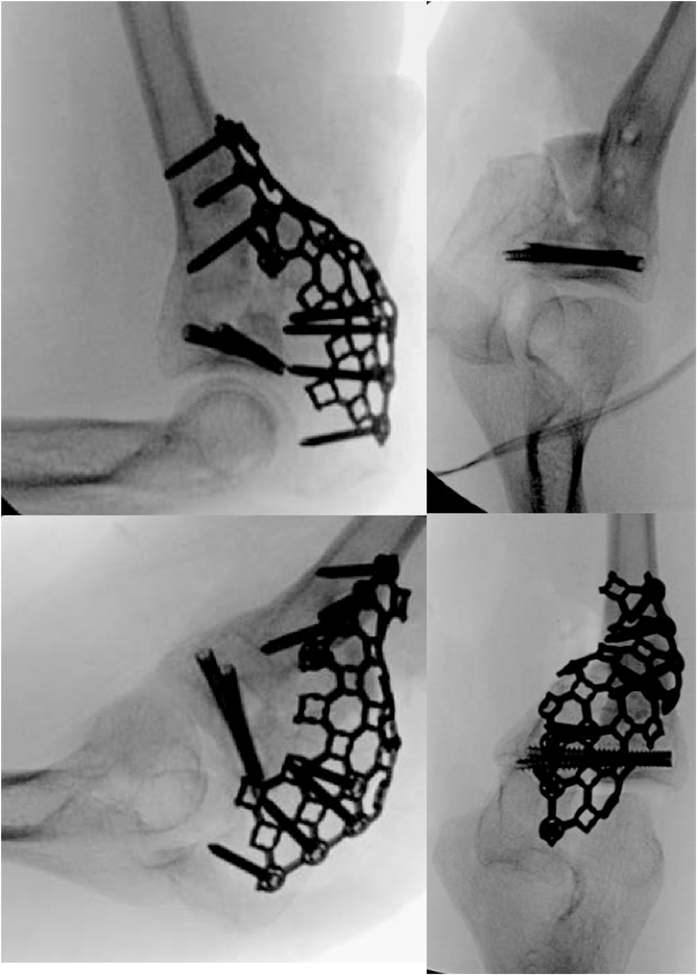
Intraoperative fluoroscopy of the one-bone forearm revision.

The previous approach was performed. All hardware was removed except the headless compression screws. The posterolateral radius and ulna were exposed and decorticated to increase the fusion area. The PRUJ was then impacted with additional iliac crest cancellous bone autograft and a tricortical iliac crest bone graft with cancellous bone graft was impacted into the posterolateral decorticated surface of the radius and ulna. A new mesh plate was contoured to allow four locking screws proximally and one cortical screw in the radius, supplemented with three locking screws to provide compression at the posterolateral fusion site (Fig. 5). The wound was closed in a layered fashion, and a posterior slab splint was placed.

### Postoperative rehabilitation

At 2 weeks, the patient was placed in a Muenster cast to allow flexion and extension but limit forearm rotation. The Muenster cast was removed, and weight bearing was advanced to a 10-pound limit.

## Results

**Figure 6:**
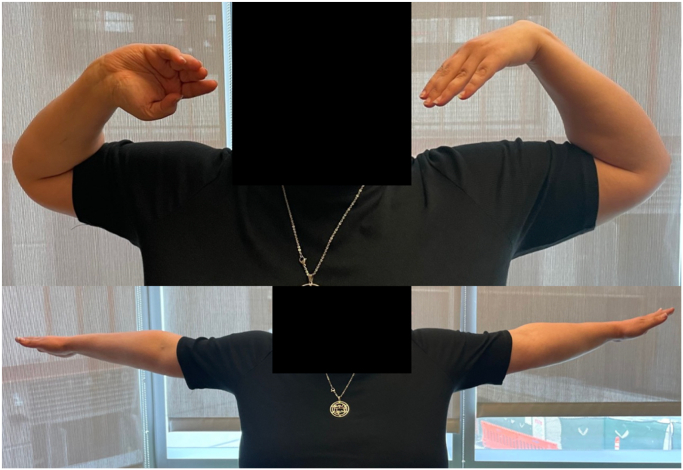
Clinical photographs at 1-year after surgery demonstrating excellent elbow range of motion.

**Figure 7:**
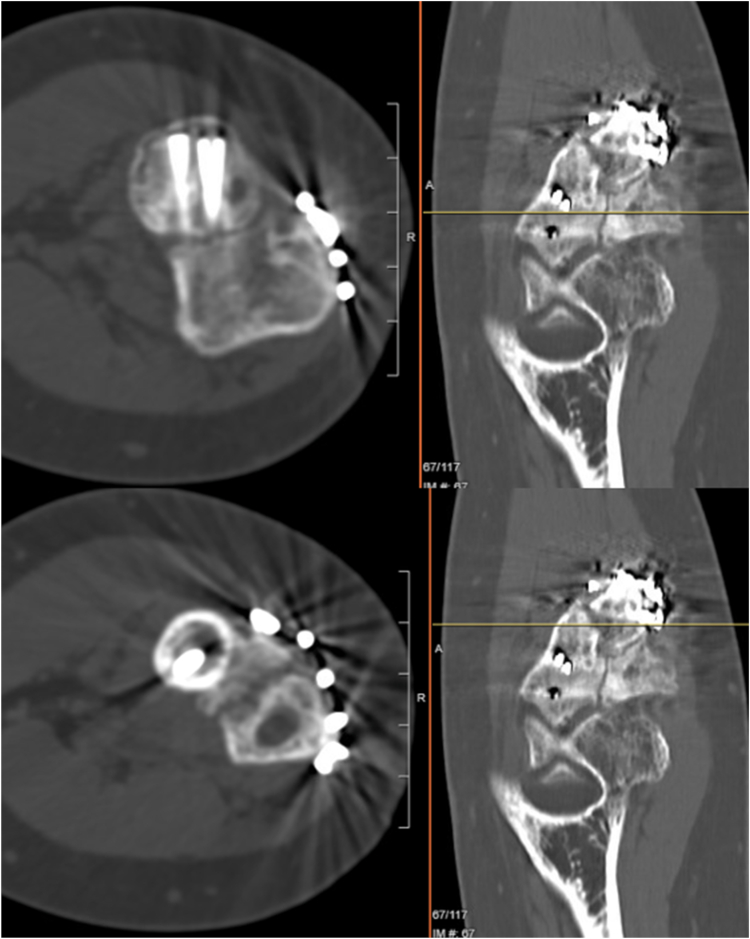
One-bone forearm revision postoperative computed tomography scan demonstrating callus formation and fusion at the revision site at 10 weeks.

**Figure 8:**
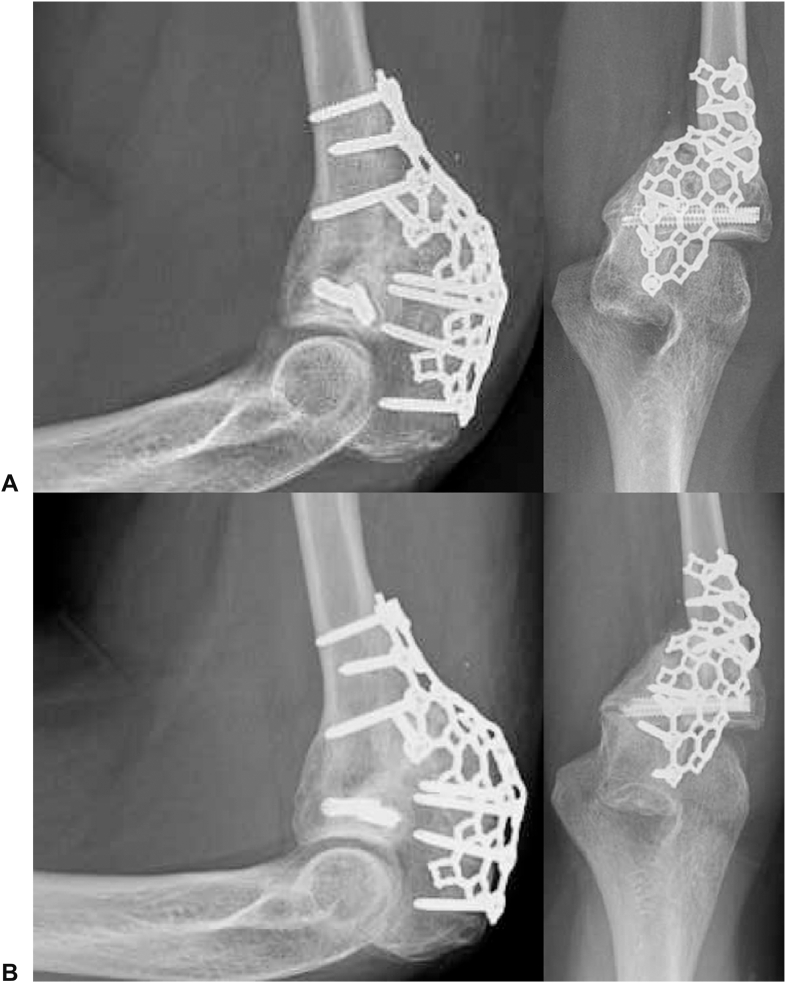
One-bone forearm reconstruction revision postoperative radiographs at **A** 10 weeks and **B** 18 months.

At 4 months, she returned full-time to her work as a baker. At 1-year postrevision, the patient’s elbow range of motion was 0° to 130° with no pain (Fig. 6). At 10 weeks, computed tomography and radiographs demonstrated interval healing of the fusion site (Figs. 7 and 8A). At 18 months, she remained pain-free with fusion across her one-bone forearm (Fig. 8B). Her Disabilities of the Arm, Shoulder and Hand score was 19.2 and Peterson score was 8/10, suggesting an excellent clinical outcome.^8^ There was no evidence of loosening of the mesh plate screws. The magnetic resonance imaging demonstrated no evidence of tumor recurrence at 2 years after resection.

## Discussion

This case demonstrates a rare presentation of ulna adamantinoma and provides a novel limb salvage surgical technique after near-complete ulnar resection. Differentiating adamantinoma from osteofibrous dysplasia is crucial. Once confirmed, initial staging studies and routine follow-up surveillance must be performed, as adamantinoma can recur more than 10 years after initial diagnosis.^1^

Most adamantinomas arise in the tibial shaft (90%),^1^ and ulnar cases are rare.^4^^,^^5^ In two large case reports of adamantinomas, both Moon et al^4^ and Keeney et al^5^ found that approximately 3% of adamantinomas occur in the ulna (6/195 and 3/85, respectively). Given the metastatic potential, the gold standard for treatment is amputation or en bloc resection with wide margins if possible.^1^ In the case presented, en bloc resection with wide margins with a one-bone forearm created a stable bony bridge between the elbow and wrist, thereby preserving the articulations of the ulnohumeral and radiocarpal joints.

The one-bone forearm was initially described by Hey-Groves^9^ in 1921 for distal radius nonunion cases that failed bone grafting procedures. Many techniques have since been described, but no consensus has been reached on optimal methodology. Devendra et al^10^ reported 38 one-bone forearm reconstructions—the largest one-bone forearm reconstruction series—consisting of various indications, used multiple techniques including plating of the proximal ulna and distal radius, free vascularized fibular reconstruction, distal radioulnar arthrodesis, and ulnocarpal fusion. Most patients had good to excellent outcomes.^10^

A distinctive feature of the case presented is the reconstruction with minimal remaining proximal ulna. Most cases describe performing synostoses both proximally and distally, but this was not an option because of the importance of obtaining negative margins for adequate tumor resection. Despite this limitation, the patient has had no difficulty with wrist function and no pain despite the lack of a distal radioulnar joint. Another noteworthy technique was the use of a trephine to core out the tract of the intramedullary nail. In general, the tract of a nail placed through a tumor is considered contaminated, so we opted to remove the tract altogether to reduce recurrence risk. By sending the trephined bone to pathology, we confirmed that our margins were truly negative. Finally, the use of a mesh plate provided a low-profile method of fixation that is highly customizable to the patient’s anatomy, essential in tumor surgery given the need for clear margins.

In summary, osteofibrous dysplasia and adamantinoma present a diagnostic dilemma. Ulnar adamantinoma may be successfully treated with en bloc resection and conversion to a one-bone forearm. In our case, a readily available mesh plate allowed customization to a variably sized remaining proximal ulna, and the absence of the distal ulna has not compromised the outcome. We recommend maximizing fusion surface area by combining the PRUJ bone grafting and compression with posterolateral radius and ulna bone grafting and compression with a tricortical iliac crest autograft with a mesh plate to decrease nonunion risk.

## Conflicts of Interest

No benefits in any form have been received or will be received related directly to this article.
